# Percutaneous and Transurethral Lithotripsy for Forgotten Ureteral Stents

**DOI:** 10.5812/numonthly.9493

**Published:** 2013-03-30

**Authors:** Ahmet Tefekli

**Affiliations:** 1 Department of Urology, School of Medicine, Bahcesehir University, Istanbul, Turkey

**Keywords:** Ureter, Stents

*Dear Editor,*

Ureteral stents are being used more commonly in the daily urological practice. They are functionally used to re-establish or maintain the patency of the ureter and are used to relieve ureteral obstruction, promote ureteral healing following surgery, and to assist with ureteral identification during pelvic surgery. Ureteral stents passively dilate the ureter; urine flows through the center of the hollow stent as well as around the stent, facilitating the passage of debris. Stent insertion initially increases ureteral peristaltic activity, but with time, the frequency and amplitude of ureteral peristalsis decreases (1, 2). Ureteral stent placement is associated with some degree of morbidity in the majority of patients that ranges from generalized urinary discomfort to urinary tract infection or obstruction. Much of the morbidity is related to the biocompatibility of the materials used to fashion the stent and, to some extent, their design; unfortunately, the ideal stent has yet to be discovered.

Despite advances in technology and communication skills, unintentionally forgotten ureteral stents represent as challenging cases, and interesting series are still being reported in the literature (3). Patients with forgotten stents may present with stone formation around stent material, severe infections, obstruction and even renal failure. Fortunately, combined endourological procedures are highly satisfactory in successful and safe management of these forgotten stents, as recently reported in the series by Rabani (4).

A detailed radiological evaluation, either with preoperative CT scans and/or preoperative ascending pyelography, is highly mandatory in the management of forgotten stents, since the stone burden at tip and the top of the stent has to be evaluated. Otherwise, aggressive traction of any part of the stent in order to remove it may cause more severe complications. Furthermore, a complete endourological set-up is necessary in the minimally invasive management of forgotten ureteral stents, including ureteroscope, nephroscope and intracorporeal lithotripters. A main element of the treatment strategy is to keep the number of interventions as low as possible (5). Although not used by Rabani, a flexible ureterorenoscope with a holmium laser for retrograde intrarenal surgery may also be very useful in the management of forgotten stents and may eliminate the need for percutaneous renal approach in order to remove the fragments in the upper urinary tract. However, it should be kept in mind that laser may be harmful to the integrity of the stent and may cause fragmentation of the stent.

Finally, as underlined by Rabani, the best treatment is prevention and it can be achieved by informing the patient in detail and programming an effective recall system (4). 
